# Calcium Flashes Orchestrate the Wound Inflammatory Response through DUOX Activation and Hydrogen Peroxide Release

**DOI:** 10.1016/j.cub.2013.01.058

**Published:** 2013-03-04

**Authors:** William Razzell, Iwan Robert Evans, Paul Martin, Will Wood

**Affiliations:** 1 School of Biochemistry, Faculty of Medical and Veterinary Sciences, University of Bristol, University Walk, Bristol BS8 1TD, UK; 2 Department of Biology and Biochemistry, University of Bath, Claverton Down, Bath BA2 7AY, UK

## Abstract

A crucial early wound response is the recruitment of inflammatory cells drawn by danger cues released by the damaged tissue. Hydrogen peroxide (H_2_O_2_) has recently been identified as the earliest wound attractant in *Drosophila* embryos and zebrafish larvae [1, 2]. The H_2_O_2_ signal is generated by activation of an NADPH oxidase, DUOX, and as a consequence, the first inflammatory cells are recruited to the wound within minutes. To date, nothing is known about how wounding activates DUOX. Here, we show that laser wounding of the *Drosophila* embryo epidermis triggers an instantaneous calcium flash, which travels as a wave via gap junctions several cell rows back from the wound edge. Blocking this calcium flash inhibits H_2_O_2_ release at the wound site and leads to a reduction in the number of immune cells migrating to the wound. We suggest that the wound-induced calcium flash activates DUOX via an EF hand calcium-binding motif and thus triggers the production of the attractant damage cue H_2_O_2_. Therefore, calcium represents the earliest signal in the wound inflammatory response.

## Highlights

► Wounding the *Drosophila* embryo epidermis leads to a rapid intracellular Ca^2+^ wave ► Blocking the Ca^2+^ response leads to reduced H_2_O_2_ and hemocytes at wounds ► DUOX’s EF hand domain is indispensible to interpret wound-induced Ca^2+^ flashes

## Results and Discussion

### Wounding the *Drosophila* Embryo Epidermis Results in an Immediate Calcium Wave

*Drosophila* embryos are able to heal laser-induced epithelial wounds [3] and in parallel mount a robust inflammatory response with the rapid recruitment of embryonic macrophages, called hemocytes [4, 5]. In *Drosophila* embryos and zebrafish larvae, hydrogen peroxide (H_2_O_2_) synthesized by the NADPH oxidase (NOX) enzyme DUOX appears to be pivotal in the stimulation of this inflammatory response [1, 2], but precisely how DUOX is activated by tissue damage remains unknown. However, DUOX possesses two canonical EF hands on an intracellular loop, which implicates cytosolic calcium as a potential regulator of H_2_O_2_ production.

To test for calcium signals in cells at the wound edge—signals similar to those observed in wounded epithelial cells in vitro [6–10], the *C. elegans* embryo epidermis, [11] or zebrafish larval tissues [12, 13]—we expressed the intracellular calcium reporter GCaMP3 [14] specifically in the epidermis via the GAL4-UAS system [15] by using the *e22c-Gal4* driver [16]. We coexpressed mCherry-moesin to visualize cortical actin in epithelial cells.

**Figure 1:**
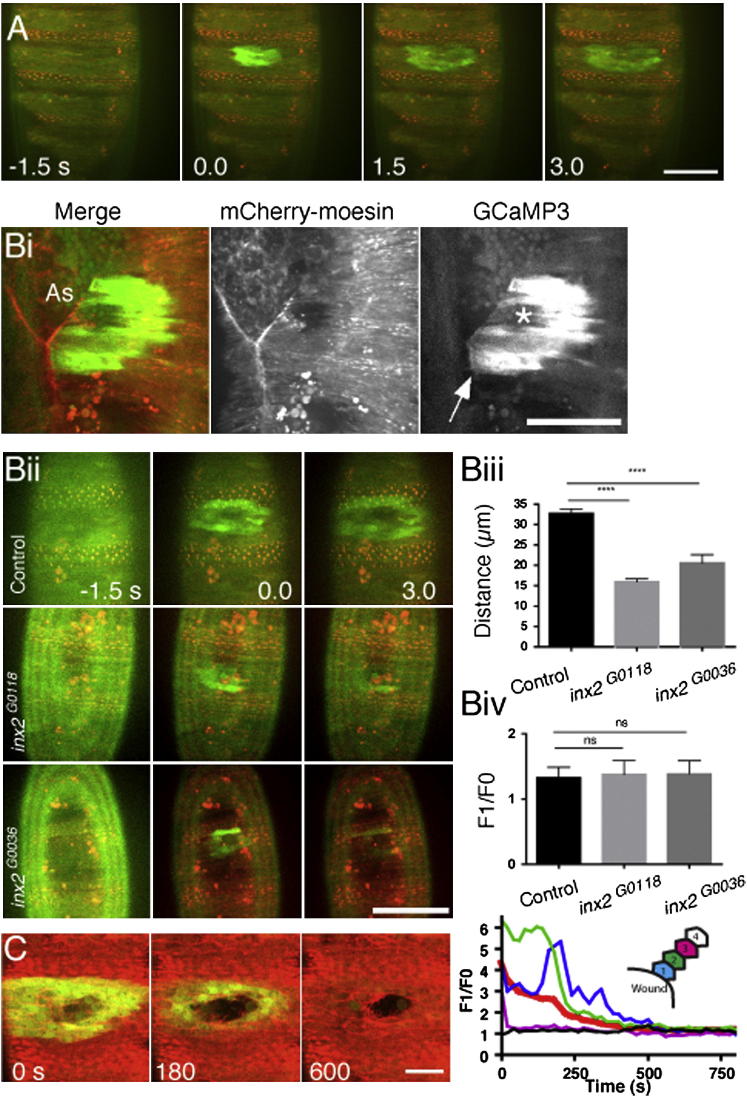
Wounding of *Drosophila* Embryos Induces an Immediate Epidermal Calcium Wave (A) mCherry-moesin (red) and GCaMP3 (green) fluorescence before and after wounding of the *Drosophila* embryonic epidermis revealed a calcium wave rapidly spreading outward from the wound margin. The scale bar denotes 50 μm, and time is in seconds. See also Movie S1. (B) In (Bi), a wound made on the dorsal epidermis in an embryo coexpressing GCaMP3 and mCherry-moesin and adjacent to the zipper front of dorsal closure shows no spread of the calcium wave from dorsal epithelium onto amnioserosa (As) or across the seam (the wound position is marked by a white star; the arrow indicates the zippering front where the calcium wave terminates). In (Bii) are stills from movies of wound-induced calcium waves in control and *inx2*^*G0118*^ and *inx2*^*G0036*^ mutant embryos; time is in seconds. Graphs in (Biii) and (Biv) reveal that the distance traveled by calcium waves, but not the initial calcium intensity (F/F0) at the wound edge, was significantly lower in *inx2* mutants than in control embryos. (A one-way ANOVA was used with a Bonferroni posttest; n ≥ 18 embryos per genotype). Scale bars in (Bi) and (Bii) denote 50 μm. Error bars in (Biii) and (Biv) represent the SEM. ^∗∗∗∗^p < 0.0001; ns = nonsignificant. (C) Resolution of the calcium wave is revealed by GCaMP3-expressing embryos (see also Movie S2). The plot shows fluorescence-intensity change normalized to background fluorescence (F1/F0) for all cells (thick red line) and cells categorized according to their position relative to the wound edge (see schematic inset for the position of cells). The scale bar denotes 20 μm, and time is in seconds.

The ventral epithelium of stage 15 embryos was laser wounded and imaged by spinning-disk microscopy. Prior to wounding, GCaMP3 fluorescence, and hence cytosolic calcium levels, did not alter in the epithelial cells; however, laser wounding resulted in a rapid calcium flash extending outward from the point of wounding as a wave to an average maximum distance of 39 ± 4.8 μm from the wound margin (SD, n = 6 movies) at a speed of 6.9 ± 2.5 μm/s (SD, n = 7 movies) across multiple cells (Figure 1A and Movie S1, available online). Ventral epithelial cells are elongated along the dorsoventral axis such that the calcium wave is propagated in a stereotypical ellipsoidal shape. Closer inspection revealed the wave to be traveling through individual cells before activating calcium release in neighboring cells (Figure S1Ai). Plotting the intensity of the GCaMP3 fluorescence in cells at varying distances from the wound edge over time showed that each cell was consecutively activated but that calcium response levels were reduced stepwise in successive rows of cells as cell distance from the wound edge increased (Figure S1Aii) until a threshold such that the wave could not travel further. This threshold was not absolute, given that larger wounds activated coordinately larger calcium waves (Figures S1B–S1D).

Previous scratch-wound analyses in cultured cells loaded with calcium reporter dyes showed that similar calcium waves depend upon extracellular diffusible mediators [8, 17]. However, in vivo, the spread of calcium is unlikely to be propagated in this manner, given that wounding the epidermis adjacent to the zippering seam of the dorsal embryonic hole, where cells are yet to be junctionally linked, resulted in immediate termination of the wave (Figure 1Bi), suggesting that cells must be intimately connected for transfer of the calcium wave. Gap junctions allow calcium waves to spread via diffusion of IP3 from cell to cell [18]. *Drosophila* gap junctions are thought to be composed of innexins, which are analogous to vertebrate connexins; therefore, to determine whether gap junctions are important for the wound-induced calcium wave to spread, we used two innexin 2 (Inx2)-null alleles, *inx2*^G0018^ and *inx2*^G0036^ [19], because Inx2 is highly expressed in the embryonic epidermis [20]. Compared to the controls, both alleles showed a significantly reduced spread of the calcium wave (Figures 1Bii and 1Biii), and the calcium influx was often restricted to the front row of cells at the wound edge. These alleles, however, did not affect the intensity of the initial calcium flash (Figure 1Biv), suggesting that innexins allow the calcium wave to propagate or maintain the calcium signal as it spreads but are not involved in its initiation.

After the rapid elevation of intracellular calcium, this signal subsequently decayed to background levels of GCaMP3 fluorescence after approximately 15 min (830 ± 360 s, n = 9 wounds; Figure 1C and Movie S2), many minutes prior to the completion of wound closure. Resolution commences in a distal-to-proximal direction, and wound margin cells return to basal levels of calcium last of all (Figure 1C and Movie S2). During the resolution period, around 40% of cells within the calcium flash zone exhibited calcium oscillations (approximately 30% oscillated only once), and no cell underwent greater than three oscillations. Cells rapidly reset their calcium machinery because subsequent wounding (within 20 min of the original wound) again elicited an identical calcium response (Figure S1E).

### Reducing the Calcium Flash Impairs the Inflammatory Response of Hemocytes

This calcium flash represents the earliest identified signal following tissue damage and might therefore orchestrate the rapid recruitment of immune cells. To assess the impact of a reduced calcium flash upon the inflammatory response, we monitored the numbers of hemocytes drawn to laser-induced epithelial wounds under a range of pharmacological and genetic treatments.

**Figure 2:**
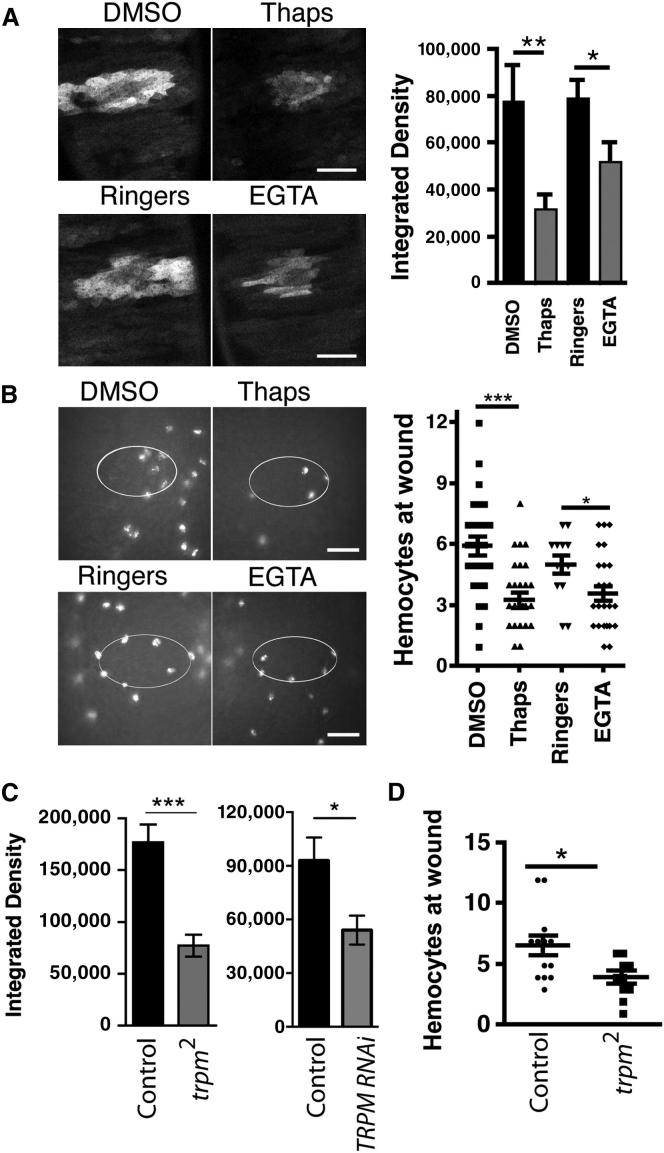
Wound-Induced Calcium Waves Activate the Inflammatory Response (A) Representative images of epithelial GCaMP3 fluorescence immediately after wounding in embryos treated with 1 μm thapsigargin or 5 mM EGTA reveal that interfering with calcium signaling impaired wound-induced calcium waves. The bar graph shows the mean integrated density of the GCaMP3 signal per embryo (n ≥ 6 embryos per treatment). The scale bars depict 25 μm. (B) Dampening calcium responses reduced recruitment of red-stinger-labeled hemocytes to wounds (wound edges are indicated by white ellipses). The scatter plot shows quantification of hemocytes per wound (the lines show the mean for ≥13 embryos per treatment). The scale bars depict 20 μm. (C) The mean integrated density of GCaMP3 fluorescence per embryo immediately after wounding of *trpm*^*2*^ mutants or embryos expressing TRPM RNAi specifically in the epithelium is lower than that for wild-type controls, indicating an epithelial role for TRPM in the generation of a calcium wave (n > 12 embryos per genotype). (D) Impairment of the calcium wave correlated with a decrease in mean numbers of red-stinger-labeled hemocytes recruited to wounds in *trpm*^*2*^ mutants (n ≥ 10 embryos per genotype). All error bars represent the SEM, and asterisks denote significance values of p < 0.05 (^∗^), p < 0.01 (^∗∗^), and p < 0.001 (^∗∗∗^) via a Student’s t test.

Treatment with 1 μM thapsigargin or 5 mM EGTA to deplete internal stores of calcium or extracellular calcium (present in the vitelline fluid surrounding the embryo at 5 mM [21]), respectively, both significantly decreased the calcium flash (as assessed by integrated density of the calcium flash zone) (Figure 2A and Figure S2A). *crq-Gal4,UAS-red stinger* transgenic embryos (which allowed us to clearly see the nuclei of hemocytes for accurate counting) were wounded after the same calcium-blocking pharmacological treatments, and the number of hemocytes present at ventral epidermal wounds was assessed after 20 min. Thapsigargin or EGTA treatments reduced the average hemocyte response to 55% or 72%, respectively, of that of negative controls (Figure 2B). Live imaging revealed no difference in the basal developmental migration of hemocytes posttreatment (Figure S2B), suggesting that the reduced recruitment was due to a failure by hemocytes to detect wounds rather than a general migration defect.

Similarly, loss-of-function mutant embryos of the TRPM channel (*trpm*^*2*^), whose *C. elegans* ortholog is important in wound-induced calcium responses [11] and is expressed across epithelial tissues in the embryo [22], displayed both a reduced calcium response after epidermal wounding (Figure 2C and Figure S2C) and reduced hemocyte migration to wounds (Figure 2D). Importantly, loss of TRPM did not perturb basal hemocyte migration or their developmental dispersal (Figure S2D). Significantly, epithelial-specific RNAi-mediated knockdown of TRPM also led to reduction in the calcium wave after wounding (Figure 2C), implicating this channel in the initial influx of calcium into and/or release of calcium from internal stores in epithelial cells.

### Calcium Activates DUOX through Its EF Hand at Wound Sites to Direct H_2_O_2_ Generation and Attract Hemocytes to Wounds

H_2_O_2_ has previously been shown to function as a wound chemoattractant for immune cells in *Drosophila* and zebrafish [1, 2] and is generated from superoxide by DUOX, a NOX enzyme [23]. More recently, DUOX has been shown to act in concert with calcium and Src family kinases to drive epithelial aspects of the regenerative processes in zebrafish larvae [13].

**Figure 3:**
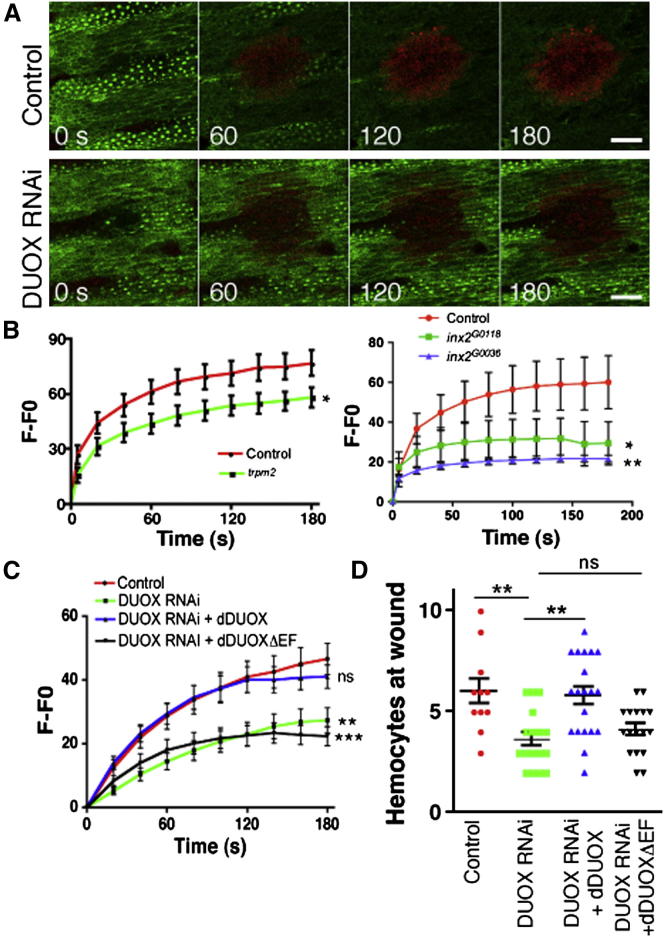
DUOX Interprets the Calcium Wave via Its EF Hand Domain to Drive H_2_O_2_ Production and Recruitment of Macrophages (A) Single confocal slices depicting GMA (actin, green) and Amplex Ultrared (H_2_O_2_, red) show that embryos ubiquitously expressing DUOX RNAi and GMA produced less H_2_O_2_ at wounds than did wild-type controls, as assayed via Amplex Ultrared fluorescence. The scale bars represent 20 μm. (B) *trpm*^*2*^ and *inx2* mutant embryos with a reduction in their wound-induced calcium responses displayed impaired H_2_O_2_ production via Amplex Ultrared (see Experimental Procedures for details of F-F0 quantification). The graphs show mean ± SEM of at least 20 (*trpm*^*2*^) and 7 (*Inx2*) embryos per genotype. (C and D) Re-expression of full-length dDUOX, but not a truncated form specifically lacking the EF hand motif (dDUOX**Δ**EF), in embryos with ubiquitous expression of dDUOX RNAi restored wound-induced H_2_O_2_ production (C) and hemocyte recruitment (D) to the levels of wild-type controls. The graphs show mean ± SEM of at least 21 (C) and 11 (D) embryos per genotype. See Movie S3 for a typical example of this data. Hemocyte numbers at wounds were determined from images of hemocytes labeled independently of Gal4 with *srp-GMA*. The p values were generated with a Student’s t test at the final time point (B [*trpm*^*2*^]), a two-way ANOVA with a Bonferroni posttest (B [*inx2*] and C), or a one-way ANOVA with a Bonferroni posttest (D). Asterisks denote p < 0.05 (^∗^), p < 0.01 (^∗∗^), and p < 0.001 (^∗∗∗^); ns = not significant.

DUOX contains a calcium-binding EF hand domain, which enables calcium to regulate the synthesis of H_2_O_2_ [24] and is therefore a good candidate for an effector of the calcium flash. H_2_O_2_ production following wounding can be visualized live by the incubation of *da-Gal4*,*UAS-GMA* (GFP-tagged actin-binding domain of moesin [25]) embryos in Amplex Ultrared, a fluorigenic reporter that is converted to its fluorescent form specifically by H_2_O_2_ in the presence of peroxidase [26] or in fixed tissue by immunospin trapping [27] (Figure S3A). Live imaging of converted Amplex Ultrared revealed a rapid accumulation of H_2_O_2_ at the wound site (Figure 3A); this accumulation was similar to that previously seen with other reporters in the *Drosophila* embryo. As previously described, H_2_O_2_ levels appeared to peak at the wound site after about 3 min [1], i.e., shortly after the calcium flash. Coexpressing a previously published DUOX RNAi [28] with *da-Gal4,UAS-GMA* showed a large reduction in Amplex Ultrared fluorescence at the wound site, confirming DUOX’s role in generating the H_2_O_2_ signal (Figure 3A). H_2_O_2_ production could not be completely abolished after DUOX RNAi expression. This could be due to either an incomplete knockdown of DUOX or the operation of alternative pathways to synthesize H_2_O_2_ (such as via other NOXs or as a consequence of cellular damage itself). As expected, expression of DUOX RNAi did not affect the calcium response (Figure S3B).

We then asked whether H_2_O_2_ production at wounds could be reduced by blockage of the calcium wave with the use of *trpm*^*2*^, *inx2*^*G0118*^, or *inx2*^*G0036*^ mutant embryos. Compared to controls, all genotypes exhibited a reduction in Amplex Ultrared signals at wound sites (Figure 3B and Movie S3), demonstrating the importance of the calcium flash in generating this potent inflammatory signal.

To test whether H_2_O_2_ production via calcium could occur in the absence of wounding, we expressed a temperature-sensitive TRPA channel by using the Gal4/UAS system. This channel opens at 37°C and induces calcium influx into cells [29], which we confirmed by coexpressing TRPA and GCaMP3 in the epidermis (Figure S3C). Treatment of TRPA-expressing embryos for 30 min at 37°C increased the levels of H_2_O_2_, as assayed via Amplex Ultrared fluorescence, suggesting that calcium influx is sufficient for the production of H_2_O_2_ in the epithelium (Figure S3D). Furthermore, overexpression of TRPA in spiracle bottle cells was sufficient to stimulate hemocyte recruitment to these sites when embryos were shifted to 37°C (Figure S3E).

To directly assess the role of calcium in the activation of DUOX, we knocked down DUOX by using RNAi and then coexpressed either full-length DUOX or a DUOX mutant lacking the calcium-binding EF hands (DUOXΔEF) [28] and monitored H_2_O_2_ production via Amplex Ultrared imaging. We were able to rescue H_2_O_2_ production in RNAi-treated embryos with full-length DUOX, but not with DUOXΔEF (Figure 3C). Furthermore, there was no difference in the numbers of hemocytes recruited to wounds in DUOX RNAi and DUOXΔEF embryos, whereas full-length DUOX restored the hemocyte response to normal levels (Figure 3D). Immunostaining and western blots using a previously generated DUOX antibody [30] revealed a knockdown of DUOX, whereas neither transgenic form of DUOX was degraded and both displayed wild-type localization within cells (Figure S3F). Taken together, these results suggest that the calcium-binding EF hands of DUOX function as a fundamental link to couple wound-induced calcium signals to the activation of DUOX and to subsequent H_2_O_2_-mediated attraction of hemocytes to wounds.

### DUOX Activity at the Wound Site Is Dependent on a DUOX Maturation Factor, NIP

In mammalian cells, the maturation factor DUOXA2/NIP is necessary for DUOX2’s folding and translocation to the plasma membrane [31]. The *Drosophila* ortholog *nip*/*DUOXA*/*mol* is expressed throughout embryogenesis and localizes to the plasma membrane during cellularization [32]. Interestingly, expression of an RNAi targeting NIP transcripts developed fragile wings highly reminiscent of those observed from overexpression of DUOX RNAi in the wing [30].

**Figure 4:**
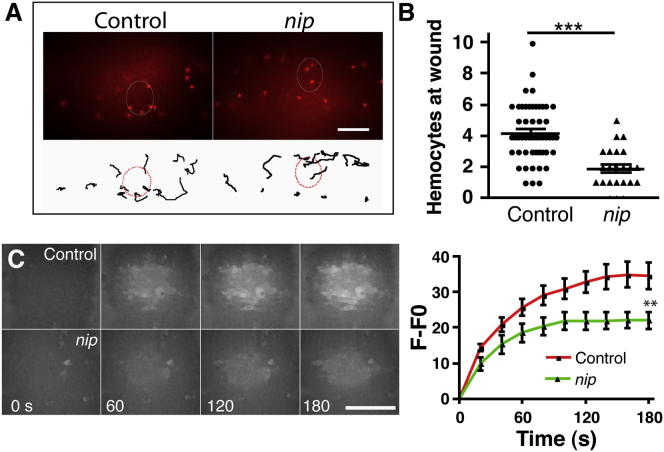
NIP Is Required for DUOX Maturation and Activation after Wounding (A) Tracking red-stinger-labeled hemocytes for 20 min after wounding revealed that fewer hemocytes migrated to wounds (indicated by white ellipses; scale bar shows 40 μm) in *nip* mutant embryos than in controls. The upper panel shows the final frame of a hemocyte movie at 20 min; the lower panel displays hemocyte tracks from the movies above and indicates that hemocytes tend to ignore wounds in *nip* mutant embryos and instead remain in their developmental positions. The wound is highlighted by the red dashed line (representative of at least three movies per genotype). (B) Quantification of mean hemocyte numbers at wounds after 20 min in wild-type versus *nip* mutant embryos; n ≥ 24 embryos per genotype. Error bars show the SEM; asterisks denote p < 0.001 (^∗∗∗^) and p < 0.01 (^∗∗^), generated via a Student’s t test. (C) Single confocal slice images of Amplex Ultrared at wounds and quantification of mean levels of fluorescence demonstrate compromised H_2_O_2_ production in *nip* mutant embryos compared to wild-type controls (see Experimental Procedures for details of quantification; n ≥ 11 embryos per genotype; scale bar shows 50 μm; time is in seconds). Error bars show the SEM; asterisks denote p < 0.001 (^∗∗∗^) and p < 0.01 (^∗∗^), generated via a two-way ANOVA with a Bonferroni posttest.

To determine whether production of the wound cue is similarly dependent upon NIP, we analyzed the inflammatory response in a previously characterized *nip*-null mutant [32]. In *nip* mutant embryos, hemocytes migrated to their appropriate developmental locations at stage 15 and lay along the midline and lateral lines of the ventral nerve cord (Figure S4A). Furthermore, hemocytes exhibited the same levels of motility as wild-type controls (Figure S4B), suggesting that they were not intrinsically affected by loss of NIP. However, live imaging revealed that hemocytes in *nip* mutant embryos often ignored laser-induced epithelial wounds (Figures 4A and 4B) such that 20 min after wounding, there were many fewer hemocytes at wounds in *nip* mutants than in wild-type embryos (Figures 4A and 4B). To test how the loss of *nip* affects DUOX function, we imaged H_2_O_2_ production in *nip* mutant embryos by using the Amplex Ultrared assay. These mutants showed a reduced H_2_O_2_ response (Figure 4C) but a normal calcium flash on wounding (Figure S4C), suggesting that NIP is critical for the wound-associated H_2_O_2_ signal. Again, we could not completely block the H_2_O_2_ signal, suggesting a contribution from maternal protein or that there are additional alternative pathways at play in the generation of H_2_O_2_ at wounds.

The importance of DUOX has been previously implicated in the synthesis of the H_2_O_2_ signal that draws immune cells to wounds [1, 2]. However, how DUOX is activated upon wounding was previously unknown. We have shown that a calcium wave, induced upon wounding the *Drosophila* embryo epidermis, leads to activation of DUOX via its canonical EF hands and the subsequent production of H_2_O_2_. Blocking wound-induced calcium flashes causes hemocytes to fail to detect wounds. We therefore believe that calcium represents the earliest key event in activation of the inflammatory response.

## Experimental Procedures

### Fly Lines and Genetics

Flies were raised and embryos were imaged at room temperature. Embryos for experiments involving RNA interference were raised at 29°C. Genotypes used are outlined in the Supplemental Experimental Procedures and Table S1.

### Imaging and Wounding

Embryos collected from overnight apple juice agar plates were dechorionated in bleach and mounted ventral side up (they were mounted dorsal side up when they were wounded near the dorsal hole) on Greiner Lumox gas-permeable culture dishes (Sigma) in halocarbon oil 700 (Sigma) or glass slides with double-sided sticky tape with voltalef oil (VWR International); see Evans et al. [33] for a video protocol. Calcium and Amplex Ultrared (Invitrogen) imaging was performed on a Zeiss LSM510 confocal microscope or a Leica DMI6000B Ultraview Vox spinning-disk system (Perkin Elmer) fitted with Micropoint nitrogen ablation lasers (Andor) for wounding; red-stinger-labeled hemocytes were imaged on a Zeiss Axioplan 2 widefield imaging system and were again wounded with a nitrogen ablation laser (Spectra-Physics). Cell tracking (manual tracking and chemotaxis plugins) and image quantification were performed with ImageJ. Prism for Mac (Graph Pad) was used for statistical analysis. For detailed description of image analyses, see the Supplemental Experimental Procedures.

### Drug and Amplex Ultrared Treatments

Dechorionated stage 15 embryos were incubated in a mixture of 1:1 heptane:drug (or Amplex Ultrared) solution in a glass vial for 30 min on a shaker. For TRPA overexpression experiments, embryos were incubated at 37°C in water prior to further treatments. Drug or Amplex Ultrared solutions consisted of *Drosophila* Ringers solution, composed of 128 mM NaCl (Fisher Scientific), 2 mM KCl (Sigma), 35.5 mM sucrose (Fisher Scientific), 5 mM HEPES (Sigma), and 4 mM MgCl_2_ (Fisher Scientific), as well as 5 mM EGTA (Sigma), 1 μM thapsigargin (1 mM stock dissolved in DMSO; Sigma), or 50 μM Amplex Ultrared. DMSO (Sigma) was added to Ringers at a 1:1,000 dilution as a negative control for thapsigargin treatments. After incubation, embryos were transferred from the heptane-aqueous interface to halocarbon oil 700 and were then mounted and imaged as above.

## Supplemental Information

Document S1. Figures S1–S4, Table S1, and Supplemental Experimental Procedures

Movie S1. Wounding Induces Calcium Waves in the *Drosophila* Embryonic Epithelium Movie showing immediate induction of a calcium wave after wounding of an embryo expressing epithelial mCherry-moesin (red) and GCaMP3 (green). An intracellular calcium wave is transmitted several cells back from the wound margin. Wounding occurs at 0 min and is marked by the white asterisk. Images taken from this movie correspond to those shown in Figure 1A. The genotype of the embryo is *w;e22c-Gal4,UAS-GCaMP3,UAS-mCherry-moesin*. The scale bar represents 50 μm.

Movie S2. Resolution of the Calcium Wave Movie showing resolution of the calcium flash in an *w;e22c-Gal4,UAS-GCaMP3,UAS-mCherry-moesin* embryo after wounding (red is mCherry-moesin, green is GCaMP3). The calcium wave is switched off in a distal-proximal direction such that the wound-margin cells extinguish their calcium response last. The scale bar represents 20 μm; time is in seconds.

Movie S3. Amplex Ultrared Live Imaging in Control versus *trpm*^*2*^ Mutant Embryos Amplex Ultrared live imaging over time in wounded control versus *trpm*^***2***^ mutant embryos shows a reduced signal when the calcium wave is perturbed after loss of TRPM. The scale bar represents 25 μm; time is in seconds.
