# Supplementary material for: Calcium Flashes Orchestrate the Wound Inflammatory Response through DUOX Activation and Hydrogen Peroxide Release
Source: Curr Biol. 2013 Mar 4;23(5):424–9. doi: 10.1016/j.cub.2013.01.058 (PMC3629559; doi:10.1016/j.cub.2013.01.058)
Supplement: Document S1. Figures S1–S4, Table S1, and Supplemental Experimental Procedures [file mmc1.pdf]

Current Biology, Volume *23*

Supplemental Information

Calcium Flashes Orchestrate the Wound Inflammatory Response through DUOX Activation and Hydrogen Peroxide Release

William Razzell, Iwan Robert Evans, Paul Martin, and Will Wood

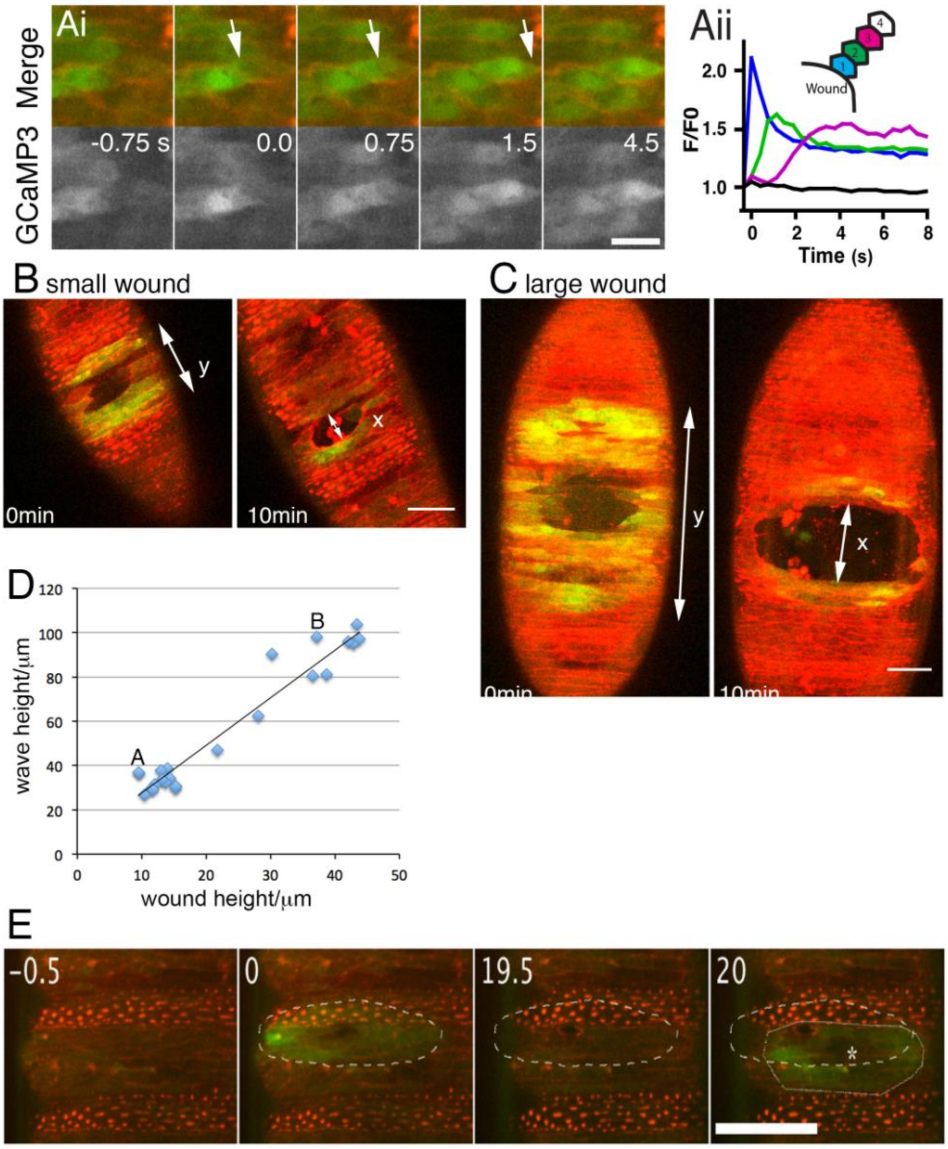

Figure S1. Larger Wounds Activate Bigger Calcium Waves and Cells at the Wound Margin Are Capable of Calcium Release after Multiple Rounds of Wounding

(Ai) GCaMP3 fluorescence indicating the intracellular calcium wave as it travels through a single cell adjacent to the wound edge (arrows indicate the leading edge of the calcium wave as it spreads through a cell).

(Aii) Plot of GCaMP3 fluorescence normalized to background fluorescence (F/F0) in cells at varying distances from the wound edge (see diagram for position of cells).

(B–C) Wounding embryos co-expressing GCaMP3 (green) and mCherry-moesin (red) in the epithelium shows a correlation between initial calcium wave and wound sizes. Maximal projections of a small (B) and large wound (C) immediately after wounding and the corresponding wound 10 min later (this was used to quantify the size of wound).

(D) Scatter plot of wound size against initial calcium wave response as quantified via initial spread of the calcium wave (wave height, x) and wound size (wound height, y); white arrows depict these measurements in (B) and (C).

(E) The epithelium is competent to activate a second wound-induced calcium wave. Images show initial wounding of embryo at 0 min (the maximum reach of the initial calcium wave is highlighted by the white dashed line) with resolution of the wave within 20 min. A second wound was made in a closely adjacent region (marked by the white asterisk); cells able to respond again are shown by the intersection of the two white ellipses.

Time is in seconds (Ai) and minutes (B,C and E); scale bars represent 10 µm (Ai), 20 µm (B-C) and 50 µm (E). Genotype of embryos is *w;e22c-Gal4,UAS-GCaMP3,UAS-mCherry-moesin*.

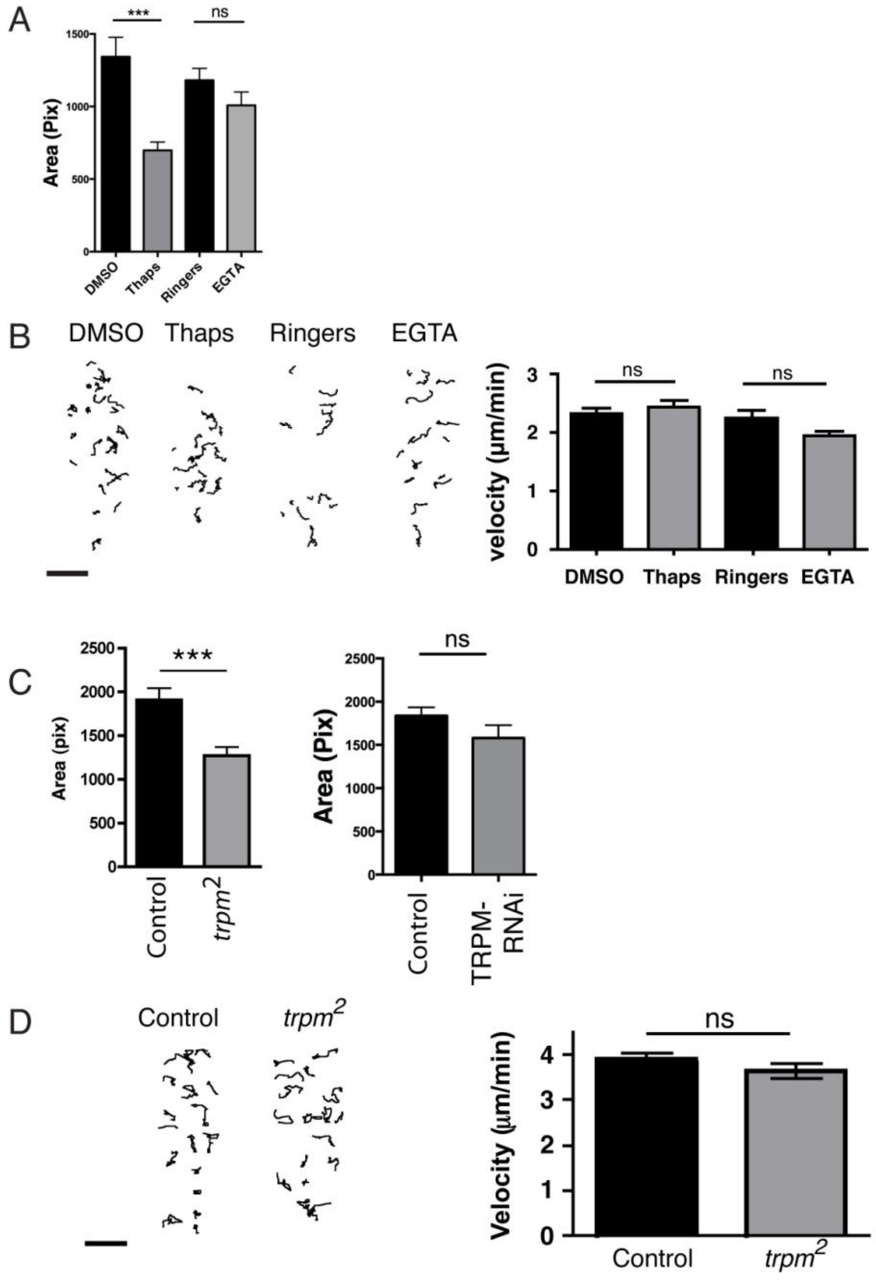

Figure S2. Pharmacological or Genetic Treatments that Reduce the Calcium Wave Do Not Affect Basal Hemocyte Migration

(A) Quantification of calcium flash area after 1 µM thapsigargin or 5 mM EGTA treatment of embryos (n ≥ 6 embryos per treatment).

(B and C) Tracks of red stinger-labeled hemocyte movement and calculation of their speeds at stage 15 after pharmacological treatment with 1 µM thapsigargin or 5 mM EGTA (B) revealed no differences compared to the appropriate controls (C) Quantification of calcium flash area in *trpm*^{2} mutant embryos (n ≥ 13 embryos per treatment) and TRPM RNAi-expressing embryos (n = 17 per genotype).

(D) Tracks of red stinger-labeled hemocyte movement and calculation of their speeds at stage 15 in *trpm*^{2} mutant embryos revealed no differences compared to the appropriate controls. Bar graphs show mean with n > 48 hemocyte tracks analyzed per genotype from at least 3 embryos.

Error bars represent the SEM; asterisks denotes p < 0.001 (***), ns = not significant (Student's t test). Scale bars represent 50 µm; genotypes are *w;;crq-Gal4,UAS-red stinger* (A and Control in D) and *w;trpm*^{2}*;crq-Gal4,UAS-red stinger* (*trpm*^{2}, D) Genotypes in A and C are the same as in Figures 2A and 2C).

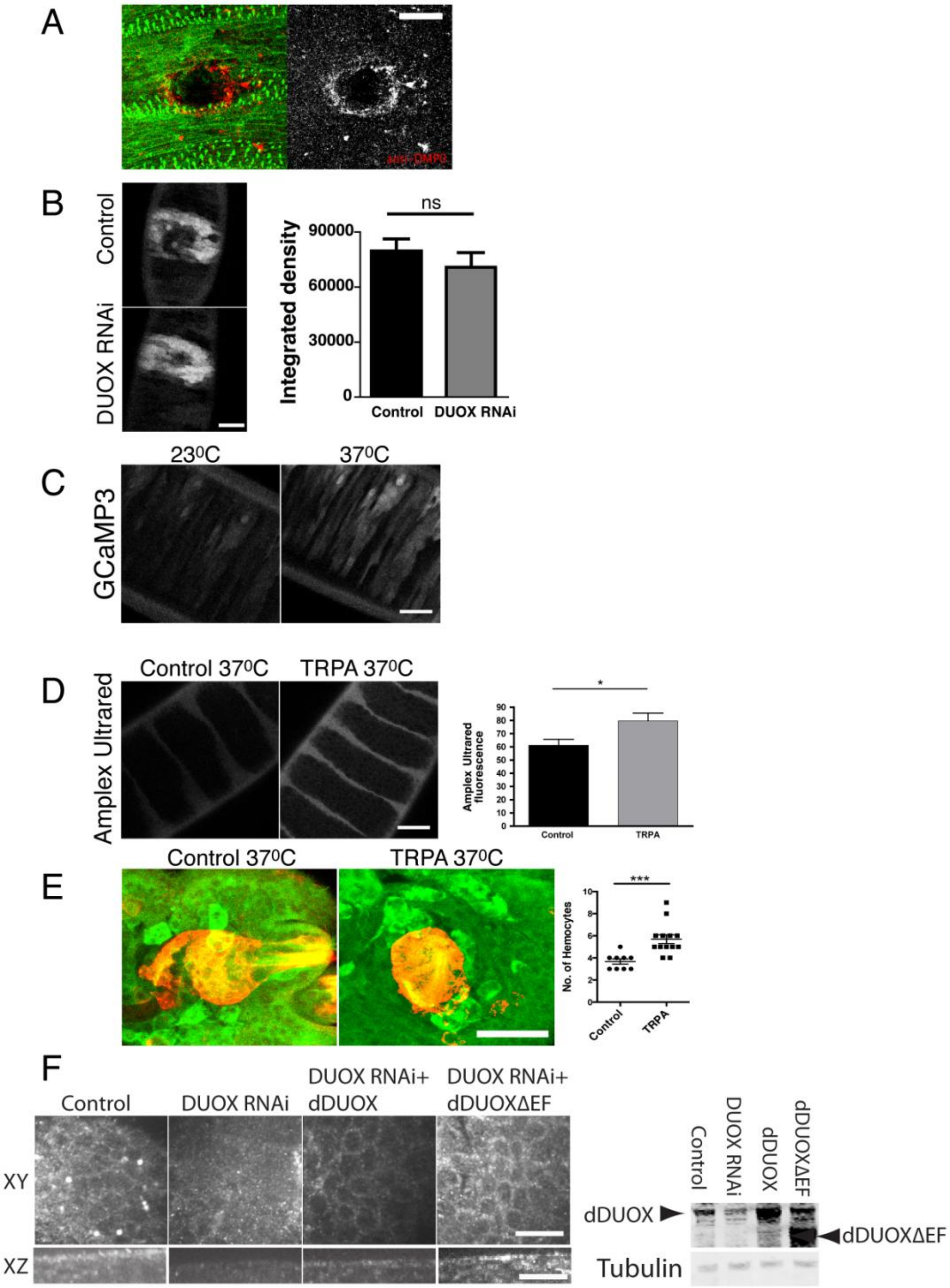

Figure S3. DUOX Knockdown Does Not Affect Wound-Induced Calcium Waves

(A) Localization of reactive oxygen species at the wound in the epidermis (green), as revealed by immune-spin trap labeling (red). Genotype is w;;da-Gal4, UAS-GMA/+. Scale bar represents 25 µm.

(B) Representative images of initial calcium waves following wounding in wild-type control and DUOX RNAi embryos. Bar graph shows mean integrated density ± SEM per embryo from ≥20 embryos per genotype. Genotypes are *w;e22c-Gal4,UAS-mCherry-moesin,UAS-GCaMP3* x *w/Y* (Control) or x *w/Y;UAS-dDUOX RNAi* (DUOX RNAi). ns = not significant via Student's t test. Scale bar represents 20 µm.

(C) GCaMP3 fluorescence with expression of TRPA via e22c-Gal4 at 23^0C or 37^0C. Genotype is *w;e22c-Gal4,UAS-mCherry-moesin/ UAS-TRPA*. Scale bar represents 25 µm.

(D) Amplex Ultrared fluorescence in control vs. TRPA-expressing embryos at 37^0C. Bar graph shows mean Amplex Ultrared fluorescence ± SEM per embryo from ≥36 embryos per genotype. Genotypes are *w;;da-Gal4,UAS-GMA/+* (Control) and *w; UAS-TRPA/+; da-Gal4, UAS-GMA/ +*. Asterisk denotes $p < 0.05$. Scale bar represents 25 µm.

(E) Hemocytes (green) were attracted to bottle cells (red) when TRPA was overexpressed using ems-Gal4. Graph showing numbers of hemocytes touching bottle cells after 30 min at 37°C ± SEM per embryo from ≥ 9 embryos per genotype. Genotypes are *w; ems-Gal4, UAS-GMA/+* (control) and *w; ems-Gal4, UAS-GMA/ UAS-TRPA* (TRPA). Asterisks denote $p < 0.05$ (*) and $p < 0.001$ (***). Scale bar represents 25 µm.

(F) Immunostaining with anti-DUOX revealed wild-type localization of both transgenic forms of DUOX (DUOX and DUOXΔEF). Immunoblotting on whole embryo lysates revealed a knockdown of DUOX protein, while neither transgenic forms of DUOX were degraded. Genotypes are the same as that for Figures 3C and 3D. For immunoblotting, genotypes are; *w;; da-Gal4, UAS-GMA/+* (control), *w; UAS-DUOX-RNAi/+; da-Gal4, UAS-GMA/+* (DUOX RNAi), *w; UAS-DUOX /+; da-Gal4, UAS-GMA/+* (dDUOX) and *w; UAS-DUOXΔEF/+; da-Gal4, USD-GMA/+* (DUOXΔEF). Scale bars represent 10 µm.

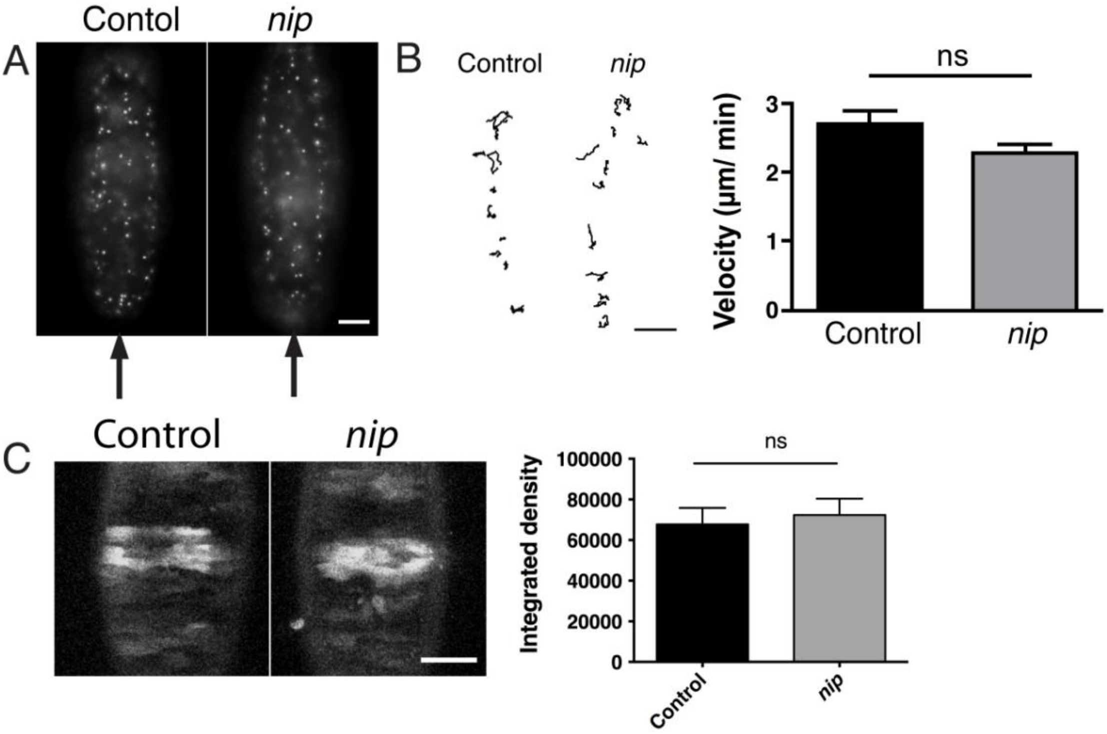

Figure S4. Genetic Knockdown of NIP Does Not Affect Hemocyte Migration

(A) Ventral views of red stinger-labeled hemocytes in wild-type control and *nip* (*mol*) mutant embryos showing hemocytes in their appropriate developmental positions along the midline and edges of the ventral nerve cord at stage 15 in both (arrows indicate ventral midline).

(B) Hemocyte tracks and mean speed ± SEM of basal migration at stage 15 (ns = not significant via Student's t test from >38 hemocyte tracks per genotype).

(C) Example images of initial calcium waves following wounding (GCaMP3, white) in wild-type control and *nip* embryos. Bar graph shows mean integrated density ± SEM per embryo from ≥14 embryos per genotype, ns = not significant via Student's t test).

Genotypes are (A) *w;;crq-Gal4,UAS-red stinger* (Control) and *w;mol^{e02670};crq-Gal4,UAS-red stinger* (*nip* mutant) and (B) *w;If/Cyo; 69B-Gal4,UAS-GCaMP3* (Control) and *w; mol^{e02670}; 69B-Gal4,UAS-GCaMP*3 (*nip* mutant). Scale bars represent 50 µm (A) and 25 µm (C)

Table S1. Genotypes of Embryos Used in This Study

Figure	Designation	Flybase gene ID	Genotype
1A,Bi,C	Control	-	*w;e22c-Gal4,UAS-mCherry-moesin,UAS-GCaMP3*
1Bii-iv	Control	-	*w/Y;e22c-Gal4,UAS-mCherry-moesin,UAS-GCaMP3/+*
1Bii-iv	inx2^{G0118}	FBgn0027108	*inx2*^{G0118}*/Y;e22c-Gal4,UAS-mCherry-moesin,UAS-GCaMP3/+*
1Bii-iv	inx2^{G0036}	FBgn0027108	*inx2*^{G0036}*/Y;e22c-Gal4,UAS-mCherry-moesin,UAS-GCaMP3/+*
2A	Drug treatments	-	*w;e22c-Gal4,UAS-mCherry-moesin,UAS-GCaMP3*
2B	Drug treatments	-	*w;ubi-DEcadherin-GFP;crq-Gal4,UAS-red stinger*
2C	Control	-	*w;If/CyO;69B-Gal4,UAS-GCaMP3*
2C	TRPM^{2}	FBgn0083959	*w;TRPM*^{2}*;69B-Gal4,UAS-GCaMP3*
2C	Control	-	*w;e22c-Gal4,UAS-mCherry-moesin,UAS-GCaMP3/+*
2C	TRPM RNAi	FBgn0083959	*w;e22c-Gal4,UAS-mCherry-moesin,UAS-GCaMP3/+;UAS-TRPM RNAi/+*
2D	Control	-	*w;;crq-Gal4,UAS-red stinger*
2D	TRPM^{2}	FBgn0083959	*w;TRPM*^{2}*;crq-Gal4,UAS-red stinger*
3A	Control	-	*w;;da-Gal4,UAS-GMA x w/Y*
3A	DUOX RNAi	FBgn0031464	*w;;da-Gal4,UAS-GMA x w/Y;UAS-dDUOX RNAi*
3B	Control (for TRPM)	-	*w;TRPM*^{2}*/CTG;69B-Gal4,UAS-GCaMP3*
3B	TRPM^{2}	FBgn0083959	*w;TRPM*^{2}*;69B-Gal4,UAS-GCaMP3*
3B	Control (for inx2)	FBgn0027108	*w/Y*
3B	inx2^{G0118}	FBgn0027108	*inx2*^{G0118}*/Y*
3B	inx2^{G0036}	FBgn0027108	*inx2*^{G0036}*/Y*
3C-D	Control	-	*w;srp-GMA;da-Gal4,srp-GMA x w/Y*
3C-D	DUOX RNAi	FBgn0031464	*w;srp-GMA;da-Gal4,srp-GMA x w/Y;UAS-dDUOX RNAi*
3C-D	DUOX RNAi + dDUOX	FBgn0031464	*w;srp-GMA,UAS-dDUOX;da-Gal4,srp-GMA x w/Y;UAS-dDUOX RNAi*
3C-D	DUOX RNAi + dDUOXΔEF	FBgn0031464	*w;srp-GMA,UAS-dDUOXΔEF;da-Gal4,srp-GMA x w/Y;UAS-dDUOX RNAi*
4A-B	Control	-	*w;;crq-Gal4,UAS-red stinger*
4A-B	NIP	FBgn0086711	*w;mol*^{e02670}*;crq-Gal4,UAS-red stinger*
4C	Control	-	*w*
4C	NIP	FBgn0086711	*w;mol*^{e02670}

Supplemental Experimental Procedures

Fly Lines and Genetics

UAS-GCaMP3 [14] and *UAS-TRPA* [29] was used to image and modulate calcium levels in the epidermis (gifts from John Gillespie and James Hodge, University of Bristol, UK). *UAS-GCaMP3* was recombined with either *e22c-Gal4,UAS-mCherry-moesin* [1] or *69B-Gal4* [34]. *crq-Gal4* [35]*,UAS-red stinger* [36] and the Gal4-independent construct *srp-GMA* [1] were used to label nuclei and the actin cytoskeleton of hemocytes, respectively. *da-Gal4* was used to express *UAS-dDUOX RNAi*, *UAS-dDUOX*, and *UAS-dDUOXΔEF* (gift from Won-Jae Lee, Seoul National University, South Korea) [28]; *UAS-TRPM RNAi* (*P{TRiP.JF01465}attP2*) [37] was expressed in the epithelium using *e22c-Gal4*. $trpm^{2}$ [22], $inx2^{G0118}$, $inx2^{G0036}$*[19]* and *nip* (mol^{e02670}) [32] mutants were obtained from Bloomington Drosophila Stock Centre (University of Indiana, IN, USA) and were balanced using *twi-Gal4* or Gal4-independent fluorescent balancers [38, 39] to discriminate homozygous mutant embryos. For a complete list of genotypes see Table S1.

Immunoblotting and Immunostaining

For immunoblotting, stage 15 embryos were carefully pierced with a glass needle in Laemmlis buffer (1 µl per embryo) and boiled for 5 min prior to separation by SDS-PAGE. Post-transfer nitrocellulose membranes were cut according to molecular weight markers and the top and lower halves incubated overnight at 4°C in guinea pig anti-dDUOX (1/500; a kind gift from Kaeko Kamei [30]) and rat anti-Tubulin (1/50000; BD Serotech), respectively. Primary antibodies were detected using IRDye 680RD (Li-Cor).

For immunostaining, dechorionated embryos were fixed in heptane:4% (anti-Fascin and spin-trap staining) or 8% PFA in PBS (anti-dDUOX) and then hand devitellinized, with the exception of embryos for Fascin staining, which were devitellinized by replacing the 4% PFA with methanol and shaking vigorously. For spin-trap immunostaining, dechorionated embryos were incubated in 50 µM DMPO (Sigma):heptane for 30 min prior to wounding and fixation. Post-fixation embryos were washed in PBS/0.1%Triton-X100, then blocked in the same solution supplemented by 1% BSA (PATx). Embryos were incubated for 2 hours at room temperature in PATx containing rabbit anti-DMPO for spin-trap staining (1/500; ALX-210-530-R050, Enzo Life Sciences), rabbit anti-GFP (1/1000; Abcam) and mouse anti-Fascin (1/50; clone sn7c, DSHB), or guinea pig anti-dDUOX (1/50). Primary antibodies were detected, post-washing, using goat anti-rabbit-TRITC (1/200; Jackson Laboratories), goat anti-mouse-FITC (1/200; Jackson Laboratories) or goat anti-guinea pig-AlexaFluor 488 (1/200; Molecular Probes) diluted in PATx.

Image Processing and Analysis

Cell tracking (manual tracking and chemotaxis plugins) and image quantification were performed in NIH ImageJ. Prism for Mac (Graph Pad) was used for statistical analysis. To quantify resolution of the calcium wave we calculated the ratio of GCaMP3 fluorescence intensity for the calcium flash zone (mean grey value taken from maximum projections of confocal images; the calcium flash zone is the maximal reach of the calcium wave) at each timepoint post-wounding compared to that of the corresponding area immediately prior to wounding (F1/F0). To quantify the initial calcium waves when comparing knockdown treatments we first subtracted background GCaMP3 fluorescence intensity (mean grey value taken from a region away from the calcium flash zone) from the intensity for the calcium flash zone from maximal projections; this value was then multiplied by the area of the calcium flash zone to give the integrated density of the calcium signal. To quantify the Amplex Ultrared signal we subtracted the background fluorescence intensity (mean grey value) from that of epithelial hole created by wounding using a single confocal slice for each time frame. This fluorescence was then compared to the initial time frame to give F-F0, except for quantification of $trpm^{2}$ vs control embryos in which the pre-wound image was instead used to calculate F0.

References

34. Wang, S.Q., Tsarouhas, V., Xylourgidis, N., Sabri, N., Tiklova, K., Nautiyal, N., Gallio, M., and Samakovlis, C. (2009). The tyrosine kinase Stitcher activates Grainy head and epidermal wound healing in Drosophila. Nature Cell Biology *11*, 890-U282.

35. Stramer, B., Moreira, S., Millard, T., Evans, I., Huang, C.Y., Sabet, O., Milner, M., Dunn, G., Martin, P., and Wood, W. (2010). Clasp-mediated microtubule bundling regulates persistent motility and contact repulsion in Drosophila macrophages in vivo. Journal of Cell Biology *189*, 681-689.

36. Barolo, S., Castro, B., and Posakony, J.W. (2004). New Drosophila transgenic reporters: insulated P-element vectors expressing fast-maturing RFP. Biotechniques *36*, 436-40.

37. Ni, J.-Q., Markstein, M., Binari, R., Pfeiffer, B., Liu, L.-P., Villalta, C., Booker, M., Perkins, L., and Perrimon, N. (2008). Vector and parameters for targeted transgenic RNA interference in Drosophila melanogaster. Nature Methods *5,* 49-51.

38. Halfon, M.S., Gisselbrecht, S., Lu, J., Estrada, B., Keshishian, H., and Michelson, A.M. (2002). New fluorescent protein reporters for use with the Drosophila Gal4 expression system and for vital detection of balancer chromosomes. Genesis *34,* 135-8 .

39. Le, T., Liang, Z., Patel, H., Yu, M.H., Sivasubramaniam, G., Slovitt, M., Tanentzapf, G., Mohanty, N., Paul, S.M., Wu, V.M., et al. (2006). A new family of Drosophila balancer chromosomes with a w- dfd-GMR yellow fluorescent protein marker. Genetics *174,* 2255-7.
