# Supplementary figures and images for: Calcium Flashes Orchestrate the Wound Inflammatory Response through DUOX Activation and Hydrogen Peroxide Release
Source: Curr Biol. 2013 Mar 4;23(5):424–9. doi: 10.1016/j.cub.2013.01.058 (PMC3629559; doi:10.1016/j.cub.2013.01.058)

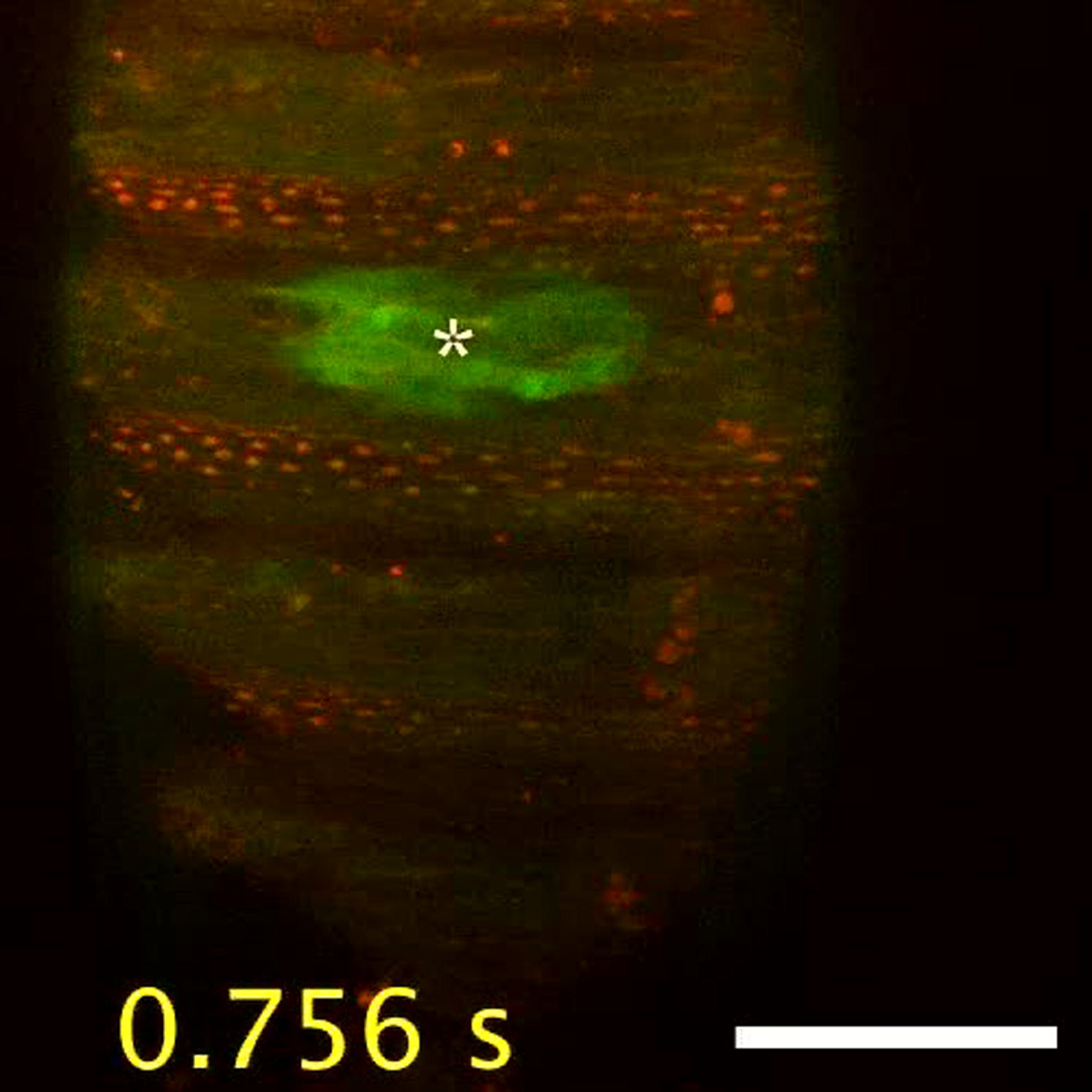

Supplement: Movie S1. Wounding Induces Calcium Waves in the Drosophila Embryonic Epithelium — Movie showing immediate induction of a calcium wave after wounding of an embryo expressing epithelial mCherry-moesin (red) and GCaMP3 (green). An intracellular calcium wave is transmitted several cells back from the wound margin. Wounding occurs at 0 min and is marked by the white asterisk. Images taken from this movie correspond to those shown in Figure 1A. The genotype of the embryo is w;e22c-Gal4,UAS-GCaMP3,UAS-mCherry-moesin. The scale bar represents 50 μm. [file mmc2.jpg]

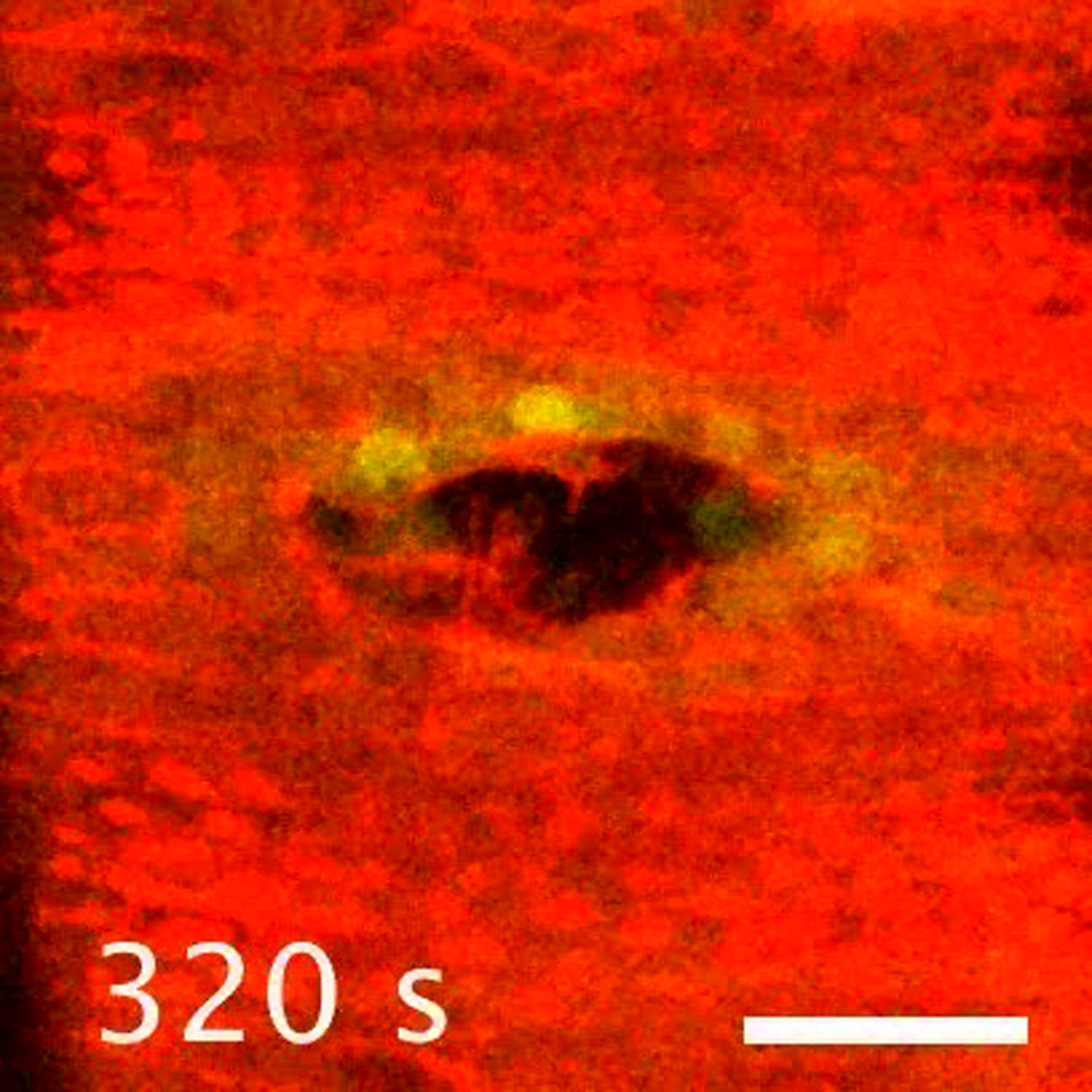

Supplement: Movie S2. Resolution of the Calcium Wave — Movie showing resolution of the calcium flash in an w;e22c-Gal4,UAS-GCaMP3,UAS-mCherry-moesin embryo after wounding (red is mCherry-moesin, green is GCaMP3). The calcium wave is switched off in a distal-proximal direction such that the wound-margin cells extinguish their calcium response last. The scale bar represents 20 μm; time is in seconds. [file mmc3.jpg]

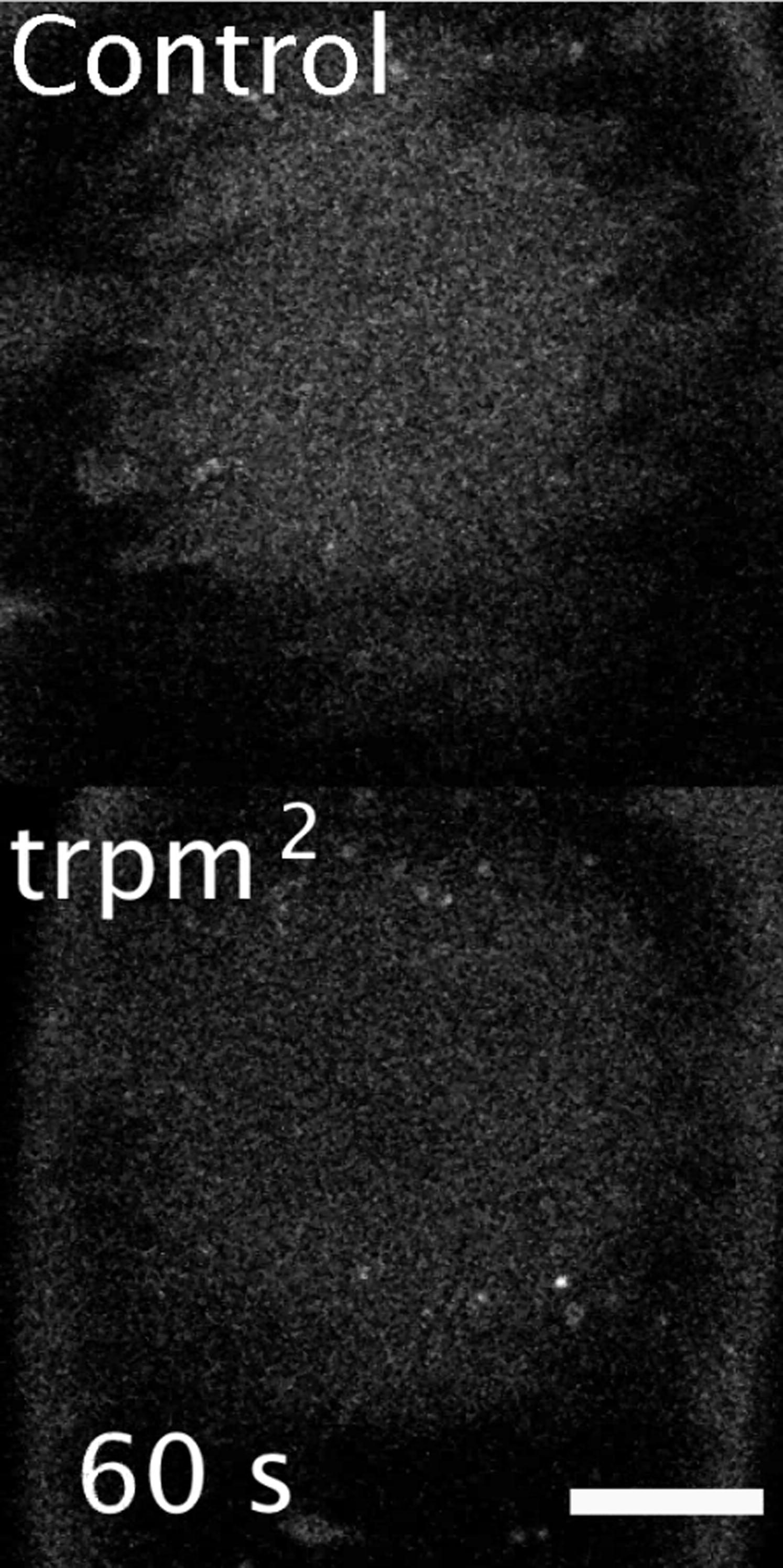

Supplement: Movie S3. Amplex Ultrared Live Imaging in Control versus trpm2 Mutant Embryos — Amplex Ultrared live imaging over time in wounded control versus trpm2 mutant embryos shows a reduced signal when the calcium wave is perturbed after loss of TRPM. The scale bar represents 25 μm; time is in seconds. [file mmc4.jpg]
